# Organizational Characteristics of Senior Centers and Engagement in Dementia-Friendly Communities

**DOI:** 10.1093/geroni/igad050

**Published:** 2023-06-09

**Authors:** Clara J Scher, Ceara Somerville, Emily A Greenfield, Caitlin Coyle

**Affiliations:** School of Social Work, Rutgers, The State University of New Jersey, New Brunswick, New Jersey, USA; Center for Social & Demographic Research on Aging, Gerontology Institute, University of Massachusetts Boston, Boston, Massachusetts, USA; School of Social Work, Rutgers, The State University of New Jersey, New Brunswick, New Jersey, USA; Center for Social & Demographic Research on Aging, Gerontology Institute, University of Massachusetts Boston, Boston, Massachusetts, USA

**Keywords:** Age-friendly, Ecological systems, Organizational capacity, Social capital, Senior centers

## Abstract

**Background and Objectives:**

Dementia-friendly communities (DFCs) are systematic and collaborative efforts to make local communities more supportive and inclusive of persons living with dementia and their care partners. This study explores how the organizational characteristics of senior centers influence their engagement in DFCs.

**Research Design and Methods:**

We used a partially mixed, concurrent, equal status design, drawing on qualitative interviews with staff from 13 senior centers leading DFC initiatives as part of a statewide dementia-friendly network in Massachusetts, as well as quantitative data from 342 senior centers collected as part of a statewide survey.

**Results:**

The qualitative results demonstrated ways in which human, social, tangible, and programmatic capital facilitate senior centers’ DFC engagement. In particular, the results illuminated the importance of social capital with organizations and groups outside of the senior center, spanning the municipal, regional, and state levels. Findings from multivariate analyses further indicated robust and strong associations between higher levels of social capital, as well as more dementia-focused programming and greater variety of funding sources, with greater likelihood of engagement in DFC work.

**Discussion and Implications:**

Results indicate the importance of policy and practice to foster both organizational capacity and multilevel systems conditions to enable and motivate senior centers’ involvement in DFC initiatives.

**Translational Significance.** Dementia-friendly communities (DFCs) constitute an innovative area of practice for communities, including senior centers as focal community-based organizations for aging in the United States. We conducted a mixed-methods study to identify organizational characteristics that facilitate engagement in DFC activities drawing on qualitative and quantitative data from senior center leaders in Massachusetts. Overall, results demonstrated how dimensions of human, social, tangible, and programmatic capital facilitate senior centers’ DFC engagement. Findings highlight the importance of social policy at multiple systems levels to more equitably and systematically encourage sufficient support for senior centers across diverse organizational and geographic contexts to engage in DFC work.

The majority of older Americans living with dementia are aging in their communities outside of institutional settings (Chi et al., 2019). To promote aging in place with dementia, researchers, service professionals, policy-makers, and advocates alike continue to emphasize the importance of strengthening community-based support for persons living with dementia and their care partners (Hebert and Scales, 2019; Heward et al., 2017; Quinn et al., 2021). There also is continued attention to advancing early detection and community prevention efforts on brain health and aging (United States Center for Disease Prevention and Control [CDC], n.d.).

The emergence of dementia-friendly communities (DFC) initiatives reflects these interests. Although there is significant variation in local implementation, DFC efforts generally emphasize reducing social stigma related to dementia (Hung et al., 2021; Mathie et al., 2022), empowering people living with dementia as they age in community (Shannon et al., 2019), and taking an assets-based approach to include the perspectives of people living with dementia in DFC development (Rahman and Swaffer, 2018). Common objectives of DFC initiatives include raising awareness about dementia among the public at large and within specific sectors, systematically enhancing local services for people living with dementia and care partners, and improving public facilities and outdoor spaces (Hebert and Scales, 2019; Shannon et al., 2019).

Senior centers are “community focal points that connect older adults to vital community services that can help them stay healthy and independent” (National Council on Aging [NCOA], n.d.). They typically provide health and wellness programming, recreational and leisure activities, and nutrition and transportation services (Laditka et al., 2012; Pardasani & Thompson, 2012). According to the NCOA, over 1 million older adults participate at senior centers across the United States daily (NCOA, n.d.). Considering the high prevalence of people living with dementia in their communities (Chi et al., 2019), and the large number of older adults who attend and benefit from senior center programming (Pardasani, 2004), senior centers are an especially relevant setting in the United States for advancing knowledge on conditions that facilitate engagement in DFC activities. Our study aims to advance a nascent research literature on DFC initiatives in the United States by using a mixed-methods research design to examine the organizational features of senior centers that influence their engagement with DFC work.

## Dementia-Friendly Communities

In the United States, the DFC movement formally began in 2009 with Minnesota’s Act on Alzheimer’s initiative, a statewide collaboration to improve the quality of care and to equip communities to better support people living with dementia (ACT on Alzheimer’s, n.d.). This development inspired several national leading organizations to launch Dementia-Friendly America (DFA) in 2015. DFA, under the administrative leadership of USAging (a national nonprofit) and with support from other groups and organizations, serves to create “a network of communities, organizations and individuals seeking to ensure that communities across the United States are equipped to support people living with dementia and their care partners” (DFA, n.d.). DFA supports its members by creating and sharing resources (e.g., DFC toolkits) and offering technical assistance (DFA, n.d.).

The scholarly literature on DFCs largely has focused on delineating the characteristics that constitute a DFC. Methodological approaches within this literature encompass reflections on practice (Lin et al., 2014; Rahman & Swaffer, 2018), as well as case studies and reports that describe the development of local DFC efforts in England, Australia, the United States, and elsewhere (Coyle, 2018; Crampton & Eley, 2013; Phillipson, 2016). In addition, there are several reviews of case descriptions, grey literature, and peer-reviewed articles that describe the characteristics of DFC efforts (Gan et al., 2021; Hebert & Scales, 2019; Hung et al., 2021; Novak et al., 2020; Shannon et al., 2019). These articles summarize strategies (e.g., drawing on multisectoral partnerships, securing funding sources) and challenges (e.g., lack of involvement of people living with dementia, difficulty gaining commitment from organizations) to DFC engagement.

To our knowledge, only one study has used formal empirical methods to examine factors associated with the development of DFC initiatives in the United States. Sun et al. (2022) conducted qualitative interviews with DFC stakeholders in both the United States and China to understand challenges and strategies during the coronavirus disease 2019 (COVID-19) pandemic. They found that despite the challenges of the pandemic, DFC leaders were able to engage people living with dementia and their care partners through partnerships with diverse community organizations, coordination of resources, and diverse funding sources. Other research using empirical methods has focused on the development of evaluation tools for DFC initiatives. For example, the DEMCOM Study—a national evaluation of DFCs in England—explored DFCs in three phases: (a) mapping DFC initiatives in England and conducting an online scoping study of DFCs (Buckner et al., 2019; Woodward et al., 2019), (b) pilot testing an existing age-friendly evaluation tool adapted for DFCs (Buckner et al., 2018), and (c) applying the evolving evaluation tool in six case study sites (Darlington et al., 2021). DEMCOM resulted in the development of a set of evaluation resources for DFCs, including an evaluation framework (e.g., thematic areas that need to be considered in evaluating DFCs), a theory of change (e.g., how DFCs can make a difference and what outcomes they can achieve), and a matrix for assessing DFC maturity (Buckner et al., 2022).

## Senior Centers in the United States

Senior centers serve to support independence and engagement as adults age within their communities (Banslaben, 2018; Cannon, 2017; Pardasani & Goldkind, 2012). Despite this common purpose, senior centers vary considerably in their organizational characteristics. Across the United States, senior centers might operate under any number of organizational structures (e.g., nonprofit, government agency), reflective of the decentralized nature of aging services and a funding structure that makes senior centers highly dependent on state, regional, local, and private sources (Bobitt & Schwingel, 2017). Furthermore, studies have documented wide variations in the professional background and pay of senior center staff (Pardasani & Sackman, 2014), funding sources and operating budget (Pardasani & Thompson, 2012), and types of programs offered (Pardasani, 2004; Pardasani & Sackman, 2014). Even within a single state, senior centers differ from each other. For example, an assessment of senior centers across Pennsylvania documented wide-ranging senior center budgets (e.g., between $17,000 and $4 million in 2017), different combinations of oversight (e.g., 66% of senior centers reported having a Board of Directors, 40% had an advisory board, and 31% reported having a center council), and variations in programming (e.g., 13% providing personal care services, 24% offering recreation activities, 80% offering evidence-based programs, and 83% providing congregate meals; Melnick et al., 2020).

## The Massachusetts Context

Massachusetts (MA) developed Dementia-Friendly Massachusetts (DFM) in 2016, with funding and partnerships involving the Massachusetts Executive Office of Elder Affairs nonprofits, and private philanthropy (DFM, 2021). DFM helps to facilitate the development of local DFC initiatives in MA and builds cohesion across state partners so that DFC concepts are rooted within existing systems of support for residents and their care partners. Cities and towns in MA join DFM by signing a pledge, with commitment from their local governments, to promote inclusivity and human dignity through supportive programming, welcoming environments, and opportunities to learn about dementia in their respective localities. The Massachusetts Councils on Aging (MCOA), a nonprofit organization, serves as the administrative backbone for DFM.

In many cases, Councils on Aging (COAs)—municipal agencies providing programs and services for older adults—serve as local leaders for DFC initiatives in MA. Municipalities in MA are mandated to have a COA by state law, and for most communities, the COA operates as a senior center. Across 351 municipalities in MA, there are 13 communities that share resources to operate a COA, yielding five regional COAs across the state for a total of 342 COAs in MA. For the purposes of this paper, we will use the term *senior centers* when referring to COAs.

## Focus of the Current Study

Our mixed-methods study focused on the following research question: How do senior center organizational characteristics influence their engagement in DFC efforts? Our study takes place in MA. The statewide emphasis on DFCs, in combination with the presence of senior centers in nearly each of the state’s municipalities, make MA an important setting for advancing research on how organizational characteristics influence the initiation and development of DFC efforts.

## Method

### Design

This study used a partially mixed, concurrent, equal status design. This design “involves conducting a study with two phases that occur concurrently” and independently, and then mixing results at the interpretation stage (Leech & Onwuegbuzie, 2009, p. 270). We used data from qualitative interviews with select senior centers and quantitative data collected from all senior centers throughout MA to explore our research question.

### Qualitative Methods

#### Sample

Representatives from the 24 communities that had signed the DFM pledge at the municipal level by March 2021 were invited by email to participate in a qualitative interview. We conducted interviews with all but four of these communities. (Invited communities that did not participate varied in their population size and median household income, and some were experiencing transitions in their DFC initiative leadership at the time of our interview request.) In addition, one community we interviewed was not included in the final sample because our discussion with the participant in the context of the interview revealed that the DFC initiative was not active. This yielded a total of 19 interviews.

For the purposes of the current study, communities were only included if the interview participants included senior center staff—yielding a total of 13 qualitative interviews. The 13 interviews were conducted with a total of 14 individuals, as one of the interviews was conducted with two individuals from the same initiative. All participants identified as non-Hispanic White (100%) and senior center directors or outreach coordinators (100%), and the majority of participants were women (93%). Table 1 provides an overview of the characteristics of the communities included in our sample, indicating their range in population size, percentage of population ages 65 and older, racial/ethnic composition, and socioeconomic status.

**Table 1.** Demographic Characteristics of Communities in the Qualitative Sample (*n* = 13)

Demographic characteristics	Mean	Min	Max
Total population size	39,589	8,548	185,428
% Population 65+	16%	9%	21%
% Population non-Hispanic White	82%	55%	98%
% Population with Bachelor’s Degree or higher	55%	30%	84%
Median household income	$103,053	$48,139	$145,679

*Note.* Data derived from the 2020 decennial Census, United States Census Bureau.

#### Data Collection

CJS and EAG conducted the semistructured interviews via Zoom, which were 90–120 min in length. The semistructured interview guide consisted of open-ended questions customized for each community based on the research team’s background preparation (e.g., review of DFC initiative websites and press releases). Questions focused on how the DFC initiative was developed and implemented. See Supplementary Section 1 for an overview of the interview guide, including sample questions commonly asked across interviews.

The study received approval from the Institutional Review Board at Rutgers, The State University of New Jersey. Participants signed an online informed consent that included information about their rights as participants and assurances regarding the confidentiality of their interview data.

#### Data analysis

All interviews were audio-recorded, transcribed, and imported into NVivo Version 12. We used techniques from grounded theory—including memo writing and iterative phases of coding—to derive insights into how organizational characteristics influence DFC engagement. Consistent with guidance from Patton (2014), we used peer debriefing and memo writing throughout all phases of the analysis to ensure credibility and rigor.

Supplementary Table 1 displays the codes and subcodes that emerged across the three iterative phases of the coding process. In summary, Phase one involved a coding shell based on sensitizing concepts derived from our analytic memos and the literature. In Phase two, CJS and EAG collaboratively subcoded the excerpts under each of the initial thematic categories and moved subcodes and excerpts originally interpreted under a preliminary theme to a different conceptual category as appropriate. Finally, in phase three, the authors recognized that the codes could be categorized according to different ecological system levels (Bronfenbrenner, 1992), with DFCs and their own organizational features at the center. In addition, at this point in the analytic process, the research team determined that the subthemes within each level of the ecosystem were aligned with existing theories about types of capital (Jackman, 2001). As a result, the subthemes were reorganized under distinct types of capital. Supplementary Table 2 provides sample excerpts and the ways they were coded across all three phases of analysis.

### Quantitative Methods

#### Sample

The quantitative data were from a comprehensive database of senior centers in MA generated through a statewide survey. The database was primarily constructed as an advocacy and program planning tool for MCOA and its members, in collaboration with the University of Massachusetts Boston, with permission to use the data for research purposes. The most recent wave of data collection was in 2020, at which time every municipal-based senior center was invited to complete three topical, web-based, surveys over a period of several months. The analytic sample for this study included MA senior centers that responded to at least one of the three survey questionnaires. Thirteen communities—representing small towns with little to no senior center capacity—were missing on all three questionnaires and were excluded from the sample. Therefore, the final analytic sample included 329 senior centers, representing 96% of all senior centers in MA.

#### Measures

##### Organizational characteristics.

Measures of senior center characteristics were selected to align with the qualitative results, with indicators of social capital (partnerships and age-friendly engagement), human capital (staffing, leadership, and volunteers), tangible capital (space, funding), and programmatic capital (service provision and variety and types of programs). Supplementary Table 3 presents additional information about each variable.

##### Level of DFC engagement.

Level of DFC engagement among senior centers was based on a single item that asked, “How would you describe your community’s progress toward becoming dementia-friendly?” Participants were asked to select from seven response options. We coded responses into three categories, including *engaged* (“My community is planning/preparing for making the community dementia-friendly” or “My community is actively making the community dementia-friendly” or “My community is maintaining dementia-friendly programs/services”), *interested* (“My community is interested in learning more about dementia-friendly”), and *nonengaged* (“My community is not thinking about dementia-friendly” or “I don’t know” or “My community tried it and gave up”). Senior centers—as community hubs for aging services—are often involved in dementia-friendly work in MA. Furthermore, MA’s statewide initiatives for age- and DFCs make awareness of DFC initiatives common knowledge throughout the Commonwealth. Therefore, we interpret the response of “I don’t know” as a lack of senior center engagement in DFC work.

#### Data analysis

We used SPSS 27. First, we estimated means and percentages for each organizational characteristic across the three levels of DFC engagement (nonengaged, interested, and engaged). We used Analysis of Variance (ANOVA) and chi-square tests to test for bivariate differences. Then, separate multinomial logistic regression models were estimated for each of the categories of capital—human, social, tangible, and programmatic—to predict the likelihood of DFC engagement. We then conducted a series of sensitivity analyses for the multivariate models to examine the robustness of the results.

## Results

### Qualitative Findings

The qualitative results yielded three thematic categories—human, social, and tangible/programmatic capital—that participants described as influencing their DFC engagement. Table 2 displays a summary of the themes under each of these categories, as described below.

**Table 2.** Summary of Key Themes from Qualitative Interviews With Senior Center Staff on Facilitators of Engagement in DFC Initiatives

Thematic category	Definition	Subthemes
Human Capital *(at the senior center)*	Senior centers utilize staff, volunteers, or interns	– Availability of staff, volunteers, or interns – Senior center leadership – Professional or personal connection to dementia
Social Capital *(at the municipal and broader systems levels)*	Senior centers leverage their connections with individuals and organizations	– Ready partner at the municipal or state level – Local government leaders are supportive – Dementia-friendly efforts supported by age-friendly elements – Regional partners’ communications platforms – Inspired by MA statewide AFC and DFC movements – Learning from other organizations about DFCs – Support to start or sustain the DFC from MA statewide organizations
Tangible and Programmatic Capital *(at the senior center)*	Senior centers utilize tangible resources (e.g., space, communications platforms) of the senior center and build from existing program offerings	– Senior center platforms (e.g., facility space, communications platforms) – Dementia-friendly programs and services already in place or in partnership with other local senior centers

*Notes*. AFC = age-friendly community; DFC = dementia-friendly community; MA = Massachusetts.

#### Human capital

Human capital broadly refers to the skills and values of actors within the senior center. A common theme under this category addressed the availability of senior center staff, volunteers, and interns in developing the DFC activity within their organizations and communities. Though some staff were paid for their time on the initiative, such as through a philanthropic grant, participants more commonly described incorporating DFC activities into primary roles in kind. For example, when asked whether there is a set number of hours allotted to work on the DFC initiative, a senior center director (Community 1) stated, “Oh no. Whatever I need, I think, it’s just part of who we are…we don’t think twice about it. We don’t say, ‘Okay, two hours and we can’t do anything.’”

Many participants described senior center staff, especially the directors, as the drivers and innovators of DFC engagement, bridging new and existing partnerships as well as creating new dementia-friendly programs that did not exist before. For example, one participant (Community 2) described how the former director—a “lifelong” resident of the community and has been with the senior center for decades—started the DFC initiative by reaching out to the mayor’s office and municipal agencies. Another participant (Community 3) stated that the DFC initiative started because the director had the initiative “ … in her wish list … She was going to be retiring soon, and she knew she wanted to accomplish a few things as part of her professional career.” Some participants also reflected on directors who were especially likely to innovate as part of their role. One director (Community 4), who started DFC work at her senior center, compared herself to the prior director as follows:

“The past director and I have two different personalities. Hers was very book-oriented, the details, accounting, all that stuff, where mine was, ‘Let’s party.’ It’s just anything new that I hear, we’re one of the first ones to do it.”

In many cases, participants described their own motivations to lead the DFC initiative because of their prior professional experience working with people living with dementia and their care partners. Several participants reflected on their work at assisted living facilities, adult day health, or running caregiver support groups as giving them insight into the experiences of aging in community with dementia and preparing them as DFC champions. In addition, some leaders were personally affected by dementia through their experience of caring for someone with cognitive impairment. For instance, one participant (Community 5) described being inspired to engage in DFC programming at her center when attending another senior center’s memory café, stating, “Having had a mother who had dementia, I thought, ‘This is something that I would have wanted to be available to me when my mom was going through that.’”

#### Social capital

Themes related to social capital refer to how senior center staff positioned their relationships with other individuals, organizations, and institutions to advance DFC work. We describe this thematic category in two parts, focusing first on local relationships and then broader systems contexts.

#### Social capital through local relationships

The most prevalent theme related to social capital at the municipal level was having local organizational partners available and ready to engage in DFC work with senior centers. Many participants described the importance of participation among local libraries, businesses, police and fire departments, municipal administration, and health care organizations to advance the DFC efforts. These partners provided facility space for, partnered on, and promoted the programs and services of the initiative. For example, one participant (Community 8) shared, “I started doing a memory café. One of the new local restaurants was very good to us, and they provided a room for it.” Other DFC leaders partnered directly with organizations on dementia-friendly programs, such as in one case where the senior center and local assisted living partnered on a new community education program (Community 7). Furthermore, some partner organizations made an effort to promote DFC programs and services throughout their local communities, such as in Community 5 where “the police chief took our brochures to different businesses to put out information about the DFC.”

Participants described support from local government officials as especially important. One way that municipal government helped the initiatives was by setting DFC-related goals for senior center staff. As one participant (Community 1) stated, “The town administrator was from a bigger city where they did [DFCs], so he wanted to make our community dementia-friendly. That’s why he set the goal for the former senior center director to create the DFC.” Another way that municipal governments assisted DFCs was by participating in the dementia-friendly action team, which further raised awareness about the cause (Community 8):

“You need a lot of cooperation and buy-in from the community. I met with our mayor and took our proposal to the city council, and the city council came on board on our committee. So we had a lot of publicity out there about what was happening.”

Some participants further described how other community initiatives and municipal committees provided a ready target for dementia-friendly advocacy. Examples included a local task force for COVID-19 response, a traffic and parking committee, equity initiatives, and a community redevelopment initiative. Participants described their efforts to embed a dementia-friendly lens within these local structures. For example, a participant (Community 7) described advocating a local transportation board to support a concierge program for accessing on-demand rides services, which was better suited for residents with memory impairment relative to “traditional” platforms for accessing these services.

In a few cases, participants stated that their communities were also advancing efforts on age-friendly community (AFC) initiatives, and the AFC-related efforts supported the DFC efforts. In one community, the DFC was a subcommittee of a larger AFC that advised the town administration on aging-related issues (Community 9). In another community, the senior center director acted as the “link” between the AFC initiative in the community and the DFC initiative at the senior center, whose representatives both would attend community health assessment meetings at the local public health department (Community 10).

Furthermore, one participant reflected on how social capital among residents helped to facilitate the DFC initiative. Describing her community as a “little village” where “everyone knows everyone” (Community 11), the participant reflected on the ease of DFC outreach in her community relative to other places, such as larger cities. In addition, some participants further described how the overall positive reputation and status of the senior center in the community helped advance the DFC initiative. For example, in Community 3, the senior center is a longstanding institution in the municipality increased the ease of forming partnerships and generated enthusiasm for the initiative: “[We get key partners to join our action team] by just inviting them after our presentations. I didn’t have to work too hard at it because our senior center has a really good reputation in our town.”

#### Social capital through broader systems contexts

Participants shared how relationships with individuals and organizations outside of their immediate geographic region also impacted their engagement in DFC activity. One prominent example was the impact of the growing age- and dementia-friendly movements taking hold in MA on the local uptake of DFCs in senior centers. Many participants shared that they learned about DFCs through the MCOA’s (statewide nonprofit) efforts to increase awareness about the age- and dementia-friendly statewide movement, with one participant stating (Community 4):

“I went to the Massachusetts COA conference and found out about dementia-friendly and age-friendly. I had just become the director and I thought, ‘I’m going to follow the lead of MCOA’ … I then proceeded to get the town involved.”

Other leaders became familiar with the DFC movement from public sector leaders, one being the governor’s commitment to making MA an age- and dementia-friendly state (Community 3): “In the State of Massachusetts, the governor made a commitment to having our state become dementia-friendly. We got the directive that this is what we want to champion over the next few years.”

In addition, some participants were motivated to launch their DFC before DFM was formed because of the Act on Alzheimer’s dementia-friendly model in Minnesota. In three such communities, senior center staff was approached by their Area Agency on Aging, which informed them about this work in Minnesota. The Area Agency on Aging sponsored the leaders of these centers to travel to Minnesota to learn more about the model and establish DFCs in their respective communities.

Furthermore, state-level organizations, largely in the private, nonprofit sector, provided nonfinancial resources to senior center leaders to start or sustain their DFC initiatives. They met with leaders in person to inform the development of the DFC initiative, facilitated the process of joining the DFM network, and distributed general information about DFCs through email and newsletters. In some cases, senior center staff received statewide technical assistance on specific programmatic components of their DFC initiatives, such as informational resources on developing memory café. Also, in some cases, state-level organizations directly partnered with DFC initiatives on programs and services within their distinct localities. Examples included individuals from state-level organizations participating in local action team meetings, partnering on local events, and administering training. For example, one community (Community 10) said, “We work closely with [name of statewide nonprofit], and one of their staff reached out to ask if we would be willing to partner on a community forum.”

#### Tangible and programmatic capital

Participants also described how tangible and programmatic capital helped to facilitate the DFC initiative. Tangible capital included communications platforms and building space. For example, participants described drawing on their organization’s longstanding communications platforms—such as email lists, newsletters, and social media—to promote DFC programs and services, raise awareness about dementia, and connect with people living with dementia and their care partners. Regarding building space, the capacity to run programs in the senior center facility was described as a convenient and cost-effective way for the senior centers to implement dementia-friendly programs, as well as a community setting in which people living with dementia and their care partners felt comfortable. As one participant (Community 6) reflected on their hosting a memory café: “We had it at a restaurant for a while, but family members and the participants really liked it at our center. It was a little bit more relaxed and open.”

Furthermore, participants capitalized on dementia-friendly programs and services that were already in place at the senior center before the establishment of the DFC initiative. One participant stated (Community 7), “We have a support group for those with dementia, and our professional staff are able to articulate what it is that people coming to the support group see as the unmet needs in the community.” These established programs provided DFC leaders with access to people living with dementia and their care partners to both learn of their experiences of aging in community, as well as to further connect them with services and supports. In addition, participants described how they partnered with neighboring senior centers in their local area to collaborate on various dementia-friendly programs and services, such as memory cafés and supportive day programs. These coordinated efforts allowed the senior centers to expand outreach to people living with dementia and caregivers in their region and to increase attendance at their dementia-friendly programs and services.

### Quantitative Findings

#### Bivariate results

Table 3 displays descriptive results of bivariate associations between each of the indicators of senior center organizational capacity and DFC engagement level. These results indicate statistically significant differences between several variables related to human, social, tangible, and programmatic capital by DFC engagement. For example, regarding social capital, DFC-engaged centers reported an average of 11.37 partnerships, compared to an average of 5.02 and 8.95 among nonengaged and interested communities, respectively. Regarding tangible capital, nonengaged centers reported ­significantly fewer funding sources (an average of 4.40 funding sources) compared to interested (an average of 5.38 funding sources) and engaged centers (an average of 6.07 funding sources). Regarding programmatic capital, centers engaged in DFC work reported offering more than twice the number of dementia-friendly programs relative to nonengaged communities. Fewer bivariate associations were found between indicators of human capital and levels of engagement in DFC work. Regarding paid staff, 65% of engaged communities reported 3 or more FTEs, significantly greater than the 40% of nonengaged communities.

**Table 3.** Descriptive and Bivariate Statistics by Level of Dementia-Friendly Engagement (Nonengaged, Interested, Engaged)

Variables	Nonengaged		Interested		Engaged			
	*n* = 95		*n* = 98		*n* = 136			
	*M*/%	*SD*	*M*/%	*SD*	*M*/%	*SD*	*F*(*df*) or χ²	
Human capital								
Has three or more FTEs on staff	40.00	49.25	55.10	49.99	65.44	47.73	7.63 (2, 203.08)	^***^
Half or more programs volunteer-run	49.47	50.26	59.18	49.40	50.00	50.19	1.23 (2, 205.20)	
MSW/LICSW on staff	21.05	40.98	28.57	45.41	35.29	47.97	2.92 (2, 209.41)	
Director							21.74 (6)	^**^
Missing or unpaid	26.32		12.24		6.62			
Makes less than $49,000	24.21		22.45		19.85			
Makes between $50,000 and $74,999	28.42		34.69		38.24			
Makes more than $75,000	21.05		30.61		35.29			
Director has been in position for 10+ years	25.26	43.68	34.69	47.84	38.24	48.78	2.33 (2, 207.84)	
Social capital								
Number of partnerships^a^	5.02	4.56	8.95	4.47	11.37	4.90	51.5 (2)	^***^
Active age-friendly	18.95	39.40	44.90	49.95	89.71	30.50	117.02 (2, 183.49)	^***^
Tangible capital								
Funding sources	4.40	2.64	5.38	2.34	6.07	2.12	13.19 (2, 196.18)	^***^
Space							13.89 (4)	^**^
No or limited space (less than 3,000 sq ft)	53.68		41.84		30.88			
Moderate space (3,000-9,999 sq ft)	25.26		34.69		33.82			
Large space (10,000 or more sq ft)	21.05		23.47		35.29			
Programmatic capital								
Dementia support programs	1.08	1.29	1.55	1.39	2.48	1.50	29.61 (2, 209.84)	^***^
Evidence-based programs^a^	1.59	2.46	2.15	2.28	2.80	2.52	7.07 (2)	^***^
Interest or leisure-based programs	4.16	3.34	6.28	2.32	7.01	1.78	29.38 (2, 179.05)	^***^
Services^a^	6.09	4.06	8.12	2.98	9.38	2.64	24.96 (2, 189.10)	^***^

*Notes*: FTE = full-time employee; LICSW = licensed independent clinical social worker; MSW = master of social work.

^a^ Welch statistics computed due to unequal variance based on Levene statistic.

^*^ *p* < .05.

^**^ *p* < .01.

^***^ *p* < .001.

#### Multivariate results

All variables related to a category of capital (human, social, tangible, or programmatic) were entered into separate blocks (Table 4). Overall, results indicated that social capital (number of partnerships and involvement in AFC initiative, Model B) as well as tangible (funding sources, Model C) and programmatic capital (dementia-friendly programming, Model D) were the most robust correlates. Regarding social capital, with a one-unit increase in the number of partnerships, senior centers were 30% more likely to be engaged in DFC work compared to nonengaged. A similar pattern emerged among interested communities such that with a one-unit increase in the number of partnerships, senior centers were 18% more likely to be interested in DFC work compared to nonengaged. Likewise, senior centers in communities with active AFC efforts were nearly 30 times more likely to be engaged and 3 times more likely to be interested in DFC initiative work compared to nonengaged. Regarding tangible and programmatic capital, with a one-unit increase in the number of funding sources, senior centers were 29% more likely to be engaged in DFC work compared to nonengaged. In addition, with a one-unit increase in the number of dementia support programs, senior centers were almost twice as likely to be engaged in DFC initiative work compared to nonengaged. Similar patterns emerged with respect to likelihood of senior centers being engaged versus interested: Increased number of partnerships, dementia support programs, and involvement in AFC work were each associated with a higher likelihood of being engaged in DFC work compared to interested.

**Table 4.** Odds Ratios for Associations between Selected Characteristics and Likelihood of Dementia-Friendly Engagement Levels (Nonengaged, Interested, Engaged) (*N* = 329)

Variables	Reference = nonengaged								Reference = interested			
	Interested OR		95% CI		Engaged OR		95% CI		Engaged OR		95% CI	
			LL	UL			LL	UL			LL	UL
Model A. Human capital												
Three or more FTEs on staff ^a^	1.370		0.664	2.826	1.772		0.895	3.505	1.293		0.675	2.480
Half or more programs volunteer-run ^b^	1.516		0.846	2.717	1.048		0.604	1.819	0.691		0.406	1.178
Social worker on staff ^c^	1.090		0.526	2.258	1.361		0.696	2.663	1.248		0.675	2.309
No paid director ^d^	0.498		0.187	1.327	0.311	*	0.114	0.848	0.624		0.215	1.811
Director makes less than $49,000 ^d^	0.933		0.396	2.202	0.892		0.398	2.000	0.956		0.436	2.096
Director makes more than $75,000 ^d^	1.128		0.514	2.475	1.045		0.505	2.162	0.927		0.481	1.783
Director tenure 10+ years ^e^	1.286		0.667	2.482	1.449		0.780	2.689	1.126		0.640	1.983
Model B. Social capital												
Partnerships	1.176	***	1.100	1.258	1.297	***	1.197	1.405	1.103	**	1.034	1.176
Active age-friendly ^f^	2.921	**	1.479	5.766	29.79	***	13.17	67.42	10.20	***	5.098	20.41
Model C. Tangible capital												
Funding sources	1.165	*	1.022	1.328	1.293	***	1.136	1.472	1.110		0.978	1.260
Moderate space ^g^	0.979		0.440	2.177	1.601		0.767	3.342	1.635		0.804	3.326
Large space ^g^	1.324		0.650	2.697	1.521		0.766	3.021	1.148		0.604	2.185
Model D. Programmatic capital												
Dementia support programs	1.182		0.855	1.635	1.918	***	1.388	2.650	1.622	***	1.227	2.145
Evidence-based programs	1.038		0.884	1.218	1.027		0.882	1.197	0.990		0.878	1.117
Interest-based programs	1.071		0.920	1.247	1.178	*	1.002	1.383	1.100		0.951	1.271
Services	1.011		0.890	1.148	1.055		0.925	1.202	1.043		0.929	1.170

*Notes*. FTE = full-time employee.

^a^Ref = Fewer than three FTEs.

^b^Ref = Less than half of programs volunteer-run.

^c^Ref = Does not have MSW/LCSW on staff.

^d^Ref = Director makes $50,000-$74,999.

^e^Ref = No director or in director in position less than 10 years.

^f^Ref = Not active in age-friendly work.

^g^Ref = No or limited space (less than 3,000 sq ft).

#### Sensitivity analyses

Three additional analyses (available upon request) were performed to test the strength and robustness of associations. First, we conducted sensitivity analyses on the sample upon excluding data from the City of Boston, given that this municipality is an outlier in terms of size relative to others. This analysis yielded no change in the magnitude or statistical significance of the results. Then, we estimated the multivariate models with measures of community-level variables from the 2019 (5-year file) American Community Survey estimates (i.e., total population, people/square mile, percent non-White, percent Latinx or Hispanic) and a measure of per capita municipal budget (2019) from the Massachusetts Department of Revenue. Community characteristics had no association with DFC engagement, and their inclusion did not alter the magnitude or strength of the associations. Lastly, we entered all of the variables that achieved significance at the bivariate level into a single multivariate model, and the results did not differ significantly from the presented findings.

## Discussion

Using a partially mixed, concurrent, equal status design, this study explored how organizational characteristics of senior centers in MA influence their engagement in DFC efforts. Qualitative findings highlighted the importance of human, social, tangible, and programmatic capital, although the quantitative findings especially highlighted the value of social capital (number of partnerships and involvement in AFC work), tangible capital (number of funding sources), and programmatic capital (dementia-centered programs). Our results in many ways support the findings of Buckner et al. (2022), which emphasize the involvement of key personnel, existing dementia-related services, and collaborations with organizations as factors that influence DFC work. We further interpret our mixed-method findings below.

### Human Capital

Qualitative and quantitative results were mixed regarding the relationship between human capital and engagement in DFC activity. The qualitative analysis revealed the importance of specific characteristics of staff members in facilitating engagement in DFC work, such as the leaders’ intrinsic motivation to be more deliberately inclusive of people aging with dementia in their community. For example, senior center staff was inspired to engage in DFC efforts due to their personal or professional connections to dementia. Prior research in more traditional dementia care settings (e.g., nursing homes and primary care memory clinics) similarly has found that providers can be motivated to engage in care for people living with dementia when they have a professional or personal connection to the cause (Sheiban et al., 2018). These results indicate ways in which drawing on human capital—beyond individuals’ skills and availability toward their intrinsic motivation to conduct the work—can facilitate senior centers’ engagement in DFC efforts.

Interestingly, although some of the quantitative variables measuring human capital (e.g., having three or more FTEs on staff) were associated with DFC engagement in the bivariate, none of the indicators of human capital were significantly associated with DFC engagement within multivariate models. Given that the qualitative results highlighted the importance of leaders’ personal characteristics (e.g., motivation to participate in DFC work and proclivity for innovation), it may be that the variables included in this study related to human capital (e.g., quantity of staff, director salary, etc.) are less salient for DFC engagement. Much of the existing literature exploring the impact of human capital on organizational capacity more broadly has focused on staff training and skill development (Douglas, 2021). Overall, our findings indicate the importance of future work to attend to multidimensional manifestations of human capital and their relationships with other organizational characteristics (e.g., partnership development, programmatic capacity) to spur innovation in senior center goals and activities.

### Social Capital

A consistent finding across the qualitative and quantitative components was that senior center engagement in DFC work stems, in part, from relationships between the senior centers and other organizations. For example, qualitative results suggested that local government officials supported DFC work by actively participating in DFC action teams at the municipal level, although at the state level, DFC initiatives were encouraged by statewide leaders’ commitment to making MA a dementia-friendly state. Similarly, multivariate results indicated that more expansive partner networks and engagement in AFC work were both distinct and strong predictors of DFC engagement.

We interpret these results in the context of the strong network of age-and dementia-friendly champions across MA that likely have facilitated senior center participation in both age-friendly and DFC work. In addition, it could be that senior centers that are participating in AFC work are also oriented to an ecosystems mindset (Fulmer et al., 2020) and therefore are well-positioned to collaborate with other organizations toward supporting people living with dementia. However, our finding that the number of senior centers’ partnerships was also independently associated with DFC engagement—regardless of senior center participation in age-friendly work—aligns with previous studies suggesting that creating innovative partnership networks across a greater ecosystem of aging-service professionals and other organizations is integral to developing DFC initiatives (Shannon et al., 2019; Sun et al., 2022).

Few studies have examined the impact of senior centers’ social capital on their organizational operations and impact. However, one related study explored the organizational ties of five senior centers in Portland, OR, and found that greater social capital among senior centers was associated with greater diversity in funding streams, advances in senior center programming, and strengthened community presence (Cannon, 2017). These findings indicate that established relationships and communications among senior centers, government departments, and other organizations—both local and beyond—are important facilitators of senior center engagement in community-centered work. This insight is especially important for DFC initiatives as explicitly collaborative, community-wide initiatives (Shannon et al., 2019). It is also consistent with findings from research on community-based organizations more broadly, which demonstrates that organizational capacity—conceptualized as “the ability of an organization to fulfill its mission” and consisting of both “financial and human resources”—is enhanced through the process of bridging social capital (Igalla et al., 2020, p. 605).

### Tangible and Programmatic Capital

Finally, qualitative findings demonstrated how senior centers drew on tangible and programmatic capital to implement DFC activities, such as facility space, communications platforms, and pre-established dementia-friendly programs and services of their own and others. The quantitative findings demonstrated that although indicators of tangible capital (e.g., facility space) and programmatic capacity (e.g., social services, professional services, nutrition) significantly differed by DFC engagement at the bivariate, those effects were diminished within multivariate analysis. Consistent with the qualitative findings, however, DFC-engaged senior centers provided significantly more dementia-focused programs (e.g., caregiver support, respite, or adult day health) than interested or nonengaged communities. Taken together, both the qualitative and quantitative results indicate that senior centers with dementia-related programming specifically—not just overall programmatic capacity—are more likely to engage in DFC initiative work.

There are several potential explanations for this finding. First, dementia is a largely invisible and highly stigmatized condition, which may serve as a barrier to the development and receptiveness of dementia-focused programming in senior centers (Bayly et al., 2020; Martin et al., 2013; Morgan et al., 2002; Riley et al., 2014). Therefore, if senior centers have leaders who are motivated to develop dementia-related programming and have the resources to do so, these leaders may also be more likely to develop DFC initiatives. Another alternative explanation is that there may be reverse causality such that creating a DFC initiative at the senior center results from the prior development of more dementia-related programming, or that senior center leaders are interpreting their dementia-related programming as evidence for their engagement in DFC work. Regardless, these results align with prior studies identifying dementia-friendly programs and services—such as support groups, respite programs, and memory cafés—as a key component of DFC efforts (Wu et al., 2019).

Lastly, we found that senior centers with greater diversity in funding sources were more likely to be engaged in DFC work compared to nonengaged. This finding is aligned with several studies highlighting the lack of funding sources as a barrier to engagement in DFC activity (Phillipson, 2016; Shannon et al., 2019; Sun et al., 2022). Although the proliferation of DFC efforts in the United Kingdom is made possible, in part, by formalized policy supports (Buckner et al., 2019; Heward et al., 2017), there have been calls for development of similar policy initiatives to increase funding for and ensure the sustainability of DFC work at the federal level in the United States (Sun et al., 2022).

### Limitations

First, the study’s setting and historical context should be acknowledged. The study was conducted exclusively in Massachusetts, with a unique state policy context that has institutionalized aging services within municipal government and that has a developed statewide network for DFC (and age-friendly) initiatives. Accordingly, the prevalence and range of qualitative themes, as well as pattern of quantitative associations, might differ in other geographic settings. Second, the dementia-friendly movement has experienced considerable growth in Massachusetts because both qualitative and quantitative data collection for this study, with over 150 communities that have formally signed the pledge to create DFC initiatives through DFM as of October 2022. Had our study used data collected even more recently, results might differ. Third, this study was conducted during the COVID-19 pandemic, a time when many aging-service organizations transitioned to crisis response, and with some centers reducing their operations and facing heightened concerns about meeting core mission activities and financing their organizations. This context has implications especially for the quantitative aspect of the study, given that survey data were collected in late 2020 during the height of the COVID-19 pandemic, although senior center directors were asked to respond to the surveys with information about operations during the 12 months prior to March 2020.

Furthermore, the qualitative component had a relatively small sample of 13 senior centers—all of which had formally committed to the DFM network as of early 2021. Themes might differ if we had included senior centers that had not formally committed to DFC efforts. In addition, our qualitative sample reflects a homogeneous composition of DFC initiative leaders, given that all participants identified as white, and most identified as women (similar to the researchers of this study). The ways DFC leaders describe engaging in DFC activity, as well as their experiences of accessing resources as part of DFC work, might vary among people belonging to other intersectional social positions. With regard to the quantitative component of the study, the sampling strategy might have resulted in biased results given that the senior centers that did not respond to the survey had very limited organizational capacity.

Lastly, the quantitative measure of DFC engagement was limited as a single-item, self-report measure. It is important for future research to incorporate other indicators—such as observational measures (e.g., updated facilities to support wayfinding) and harmonized responses regarding engagement across multiple reporters—to add depth to the measurement of DFC engagement. In addition, given that this study included variables focusing on organizational characteristics that facilitate DFC efforts, future qualitative research should explore factors that might prevent an organization from engaging in DFC work altogether.

## Conclusion

In sum, this partially mixed-methods study found that organizational characteristics related to human, social, tangible, and programmatic capital facilitate senior center DFC engagement. In particular, our findings emphasize the importance of multisystem supports for senior centers to foster the development of partnerships with other organizations and dementia-friendly programs, as well as considerations of organizational, municipal, and broader systems-level capacity, to expand and strengthen DFC efforts. These results can inform the development of formal policy supports to facilitate DFC work in senior centers. Optimizing such investments for the greatest impact likely will require coordination and cooperation from local, regional, and state entities, as our findings demonstrate how senior centers engaged in DFC efforts were able to enhance their organizational capacity by leveraging relationships with both municipal and statewide partners. Finally, our results suggest the importance of developing a theory to explain the multidimensional causal processes through which organizational characteristics influence DFC engagement, as well as how such engagement might reciprocally expand capacity in other related areas. Such research can include longitudinal, qualitative designs that prospectively study trajectories of DFC work, as well as more advanced multivariate techniques that can model greater complexity in dynamic inter-relationships across contexts. Continued research on what leads diverse organizations and communities to engage in DFCs over time is important for situating this work within broader literatures on innovations in aging, especially with respect to community-based organizations as a leverage point (Buckner et al., 2022).

## Supplementary Material

igad050_suppl_Supplementary_Material
