# Supplementary material for: Organizational Characteristics of Senior Centers and Engagement in Dementia-Friendly Communities
Source: Innov Aging. 2023 Jun 9;7(5):igad050. doi: 10.1093/geroni/igad050 (PMC10374275; doi:10.1093/geroni/igad050)
Supplement: igad050_suppl_Supplementary_Material [file igad050_suppl_supplementary_material.docx]

**Online Supplementary Material**

**Section 1: Overview of the Semi-Structured Interview Guide**

The interview guide was developed as part of a study on the implementation of DFC initiatives in Massachusetts. All interviews included six main segments, as outlined below. Responses to the questions in segments #2, #3, and #4 were of most direct relevance to the current study’s research question regarding organizational characteristics and senior centers’ engagement in DFC work. We prepared initiative-specific questions under each segment based on background information about the initiatives from our review of initiative-specific promotional information, such as newsletters, websites, and event flyers, which were available to the public. Listed below are examples of questions under each segment of the interview.

**Segment #1: Introduction and Background Questions**

- How did the idea of having a dementia-friendly community initiative first come about?
- What do you think motivated people to come together in this way? For example, had people in your community worked together on dementia in the past?
- We see that your community became pledged as part of DFM on [DATE]. Can you explain the timing of that development?
- How would you describe the connection between your community’s dementia-friendly and age-friendly efforts (if relevant)?

**Segment #2: Programmatic Emphasis**

- Has your DFC engaged in any of the following activities? Explain.
  - Educating the public and key community leaders, such as through trainings on dementia awareness or healthy brain aging.
  - Enhancing local services and supports for people living with dementia and their care partners, such as by creating easier access, greater variety, better quality, or more coordinated services.
  - Making the physical infrastructure more accommodating of people living with dementia and their care partners, such as through improving mobility and transportation services, outdoor spaces, and housing.

**Segment #3: Leadership and Action Team**

- How is the DFC initiative staffed? Do you have a formal number of hours a week that you are allowed to work on the DFC initiative as part of your role at the senior center?
- What would you say is the purpose of the action team? What do you see as the value added of working together? Are there any examples of how the group has accomplished something that the senior center alone would not have been able to?
- What has been the experience of engaging residents with cognitive impairment or dementia involved as part of the action team?
- What has been your experience of engaging residents from historically marginalized groups such as by race or ethnicity towards dementia-friendly goals?

**Segment #4: Sources of Support**

- We are going to share a list of organizations that aim to support dementia-friendly communities throughout Massachusetts. Can you reflect on them and ways in which they might have helped your work?

**Segment #5: Pandemic Effects**

- Do you think having the DFC initiative made any difference in the efforts to respond to crises?
- We have heard from other communities that the pandemic slowed down dementia-friendly community plans and activities. Has there been any ways in which the pandemic has slowed down your work?
- Others have shared with United States that in some ways, the pandemic has brought about new opportunities to engage in this work. Are there any examples of how the pandemic has enhanced your efforts?

**Segment #6: Future Goals and Conclusion**

- Can you share with United States broadly what you see as your most important action steps moving forward with the DFC initiative?
- Is there anything else that you would like to share about your experiences?

**Section 2: Supplementary Tables**

**Supplementary Table 1. Three Phrases of Iterative Code Development for the Qualitative Analysis**

**Supplementary Table 2. Three Sample Interview Excerpts and How They Were Coded across Three Iterations of Coding**

| **Excerpt** | **Iteration 1 Code** | **Iteration 2 Code** | **Iteration 3 Code** |
| --- | --- | --- | --- |
| “We had our own memory café at a restaurant for awhile, but family members really liked it in our center because it was a little bit more relaxed and open.” | *DF Programs and Services* | *Senior Center Facility Space as a Platform* | *Senior Center / Tangible/Programmatic Capital / Facility Space* |
| “I think the town administrator [had done dementia-friendly] in another community. That’s why he gave it to the former director as one of her goals.” | *Local Government* | *Local Government Context / Government Leaders are Supportive* | *Municipal Context / Social Capital/ Local Government Leaders are Supportive* |
| “[Name of non-profit leader] maintains a great list of appropriate entertainers [for memory cafes]. The list of speakers gave us some different ideas for entertainers.” | *Technical Support from Statewide Organizations* | *External Contexts / Support to Start or Sustain from MCOA or others* | *Broader Systems Influences / Social Capital/ Support to Start or Sustain from MA statewide partners* |

**Supplementary Table 3. Measures of Senior Center Characteristics in the Quantitative Analysis**

| **Measure** | **Type** | **Description** |
| --- | --- | --- |
| **Human Capital** | | |
| Number of full-time equivalents (FTEs) | Binary | - 1=Employs three or more FTEs - 0=Fewer than three FTEs |
| Volunteer-run programs | Binary | - 1=Half or more programs are run by volunteers - 0=Fewer than half of programs run by volunteers |
| Social worker (MSW or LICSW) on staff | Binary | - 1=Yes - 0=No |
| Director | Categorical | - 1=Unpaid or not reported - 2=Earns less than $49,000 a year - 3=Earns between $50,000 and $74,999 a year - 4=Earns more than $75,000 a year |
| Director Tenure | Binary | - 1=Current director has been in position for 10 or more years - 0=Fewer than 10 years or not reported |
| **Social Capital** | | |
| Partnerships | Discrete | - Number of formal partnerships reported (e.g., schools, emergency medical services, non-profits, other senior centers), ranging from 0 to 22 |
| Active age-friendly | Binary | - 1= Active communities: “My community is planning/preparing for making the community age-friendly, ""My community is actively making the community age-friendly," or "My community is maintaining age-friendly programs/services." - 0=Not engaged communities: “My community is interested in learning more about age-friendly” “I don’t know,” “My community is not thinking about age-friendly issues,” “My community tried it and gave up,” or not reported |
| **Tangible Capital** | | |
| Size of space | Categorical | - 1= No dedicated space or less than 3,000 square feet - 2=Moderate space: Occupies 3,000-9,999 square feet - 3=Large space: Occupies 10,000 or more square feet |
| Funding sources | Discrete | - Number of funding sources reported (e.g., state allotment, municipal budget, philanthropic grants), ranging from 0 to 11 |
| **Programmatic Capital** | | |
| Dementia support programs | Discrete | - Number of dementia support programs reported (i.e., memory café, memory screening, caregiver support, Alzheimer’s or dementia support, operating an adult day program, providing access to adult day programs), ranging from 0 to 6 |
| Evidence-based programs | Discrete | - Number of evidence-based programs offered (e.g., evidence-based Tai Chi, Chronic Disease Self-Management, A Matter of Balance, Aging Mastery program), ranging from 0 to 14 |
| Interest-based programs | Discrete | - Number of interest or leisure-based programs (e.g., cards, arts and crafts, billiards) provided, ranging from 0 to 11 |
| Services | Discrete | - Number of services offered (e.g., information and referral, case management, application assistance, health insurance counseling, mental health services, hoarding efforts, tax preparation, legal services, regular lunch, transportation), ranging from 0 to 15 |
